# Beyond What Meets the Eye: Imaging and Imagining Wood Mechanical–Structural Properties

**DOI:** 10.1002/adma.202001613

**Published:** 2020-08-23

**Authors:** Eleni Toumpanaki, Darshil U. Shah, Stephen J. Eichhorn

**Affiliations:** ^1^ Bristol Composites Institute CAME School of Engineering University of Bristol University Walk Bristol BS8 1TR UK; ^2^ Department of Architecture Centre for Natural Materials Innovation University of Cambridge Cambridge CB2 1PX UK

**Keywords:** diffraction, imaging, spectroscopy, structure–property relationships, wood

## Abstract

Wood presents a hierarchical structure, containing features at all length scales: from the tracheids or vessels that make up its cellular structure, through to the microfibrils within the cell walls, down to the molecular architecture of the cellulose, lignin, and hemicelluloses that comprise its chemical makeup. This structure renders it with high mechanical (e.g., modulus and strength) and interesting physical (e.g., optical) properties. A better understanding of this structure, and how it plays a role in governing mechanical and other physical parameters, will help to better exploit this sustainable resource. Here, recent developments on the use of advanced imaging techniques for studying the structural properties of wood in relation to its mechanical properties are explored. The focus is on synchrotron nuclear magnetic resonance spectroscopy, X‐ray diffraction, X‐ray tomographical imaging, Raman and infrared spectroscopies, confocal microscopy, electron microscopy, and atomic force microscopy. Critical discussion on the role of imaging techniques and how fields are developing rapidly to incorporate both spatial and temporal ranges of analysis is presented.

## Graphical abstract

A review of the imaging of wood at various length scales is presented, showing how hierarchical properties are achieved and demonstrating unique levels of physical and mechanical performance.

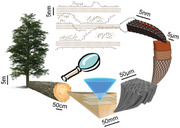

## 1 Introduction

**Figure 1:**
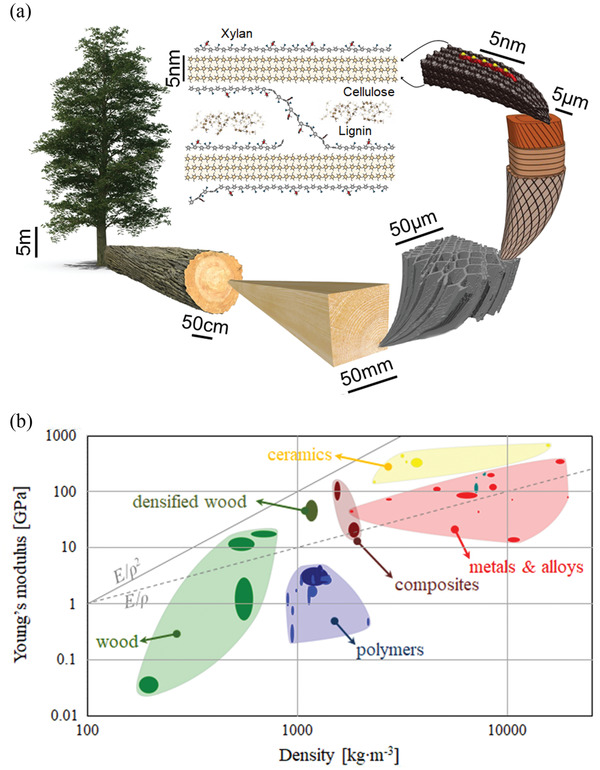
a) Schematic of the length scales of wood—from the size of trees themselves (many meters high) down to the molecular scale of the components of the wood cell wall, e.g., cellulose, hemicellulose (xylan for dicots) and lignin. Image of wood tissue, a wood cell, and a cellulose microfibril with a short xylan chain, and the generic structural arrangement of secondary cell wall polymers (labeled as cellulose, xylan, and lignin). b) Asbhy plot showing the relationship between Young's modulus of different materials and their densities—comparing native woods and densified woods,^[^ ^19^, ^20^ ^]^ with polymers, composites, and metals and alloys. Lines show the relationships between Young's modulus (*E*) and density (ρ) with *E*/ρ^2^ (solid line) and *E*/ρ (dotted line). Image of wood tissue: Adapted with permission.^[^ ^16^ ^]^ Copyright 2018 The Authors, published by the Royal Society. Image of wood cell: Adapted with permission.^[^ ^17^ ^]^ Copyright 2018, Elsevier. Images of cellulose microfibril and the generic structural arrangement of secondary cell wall polymers: Adapted under the terms of the CC‐BY Creative Commons Attribution 3.0 Unported license (https://creativecommons.org/licenses/by/3.0/).^[^ ^18^ ^]^ Copyright 2014 The Authors, published by Society for Experimental Biology and John Wiley & Sons Ltd.

Wood is one of the oldest materials to have been used for the construction of dwellings, ships and tools, among many other ancient applications. Indeed, the word “material” itself derives from the Latin for “trunk of a tree.” The material wood is in fact a composite, comprising many chemical (cellulose, lignin, and hemicellulose) and structural components on large (one to thousands of micrometers) and small (three to hundreds of nanometers) length scales (see **Figure** **1**a). Its hierarchical composite structure renders wood with excellent mechanical, transport, and optical properties, unparalleled in both other natural and many man‐made materials.

At the micrometer scale wood presents itself as a cellular solid (Figure 1a), and at the nanometer scale it has a structure that strongly influences mechanical properties. Cellulose, hemicellulose, and pectin are the main polysaccharides in wood consisting of more than 70% of the dry content,^[^ ^1^ ^]^ with cellulose being responsible for 40–50% of the dry weight.^[^ ^2^ ^]^ The concentration of the wood polymer components and their structural arrangement within the cell wall varies between softwood and hardwood species. The cell wall composition reflects the response of wood to environmental, mechanical conditions, and degradation over its lifetime.^[^ ^3^ ^]^

Cellulose comprises β‐glucose units that are covalently linked in the 1,4 positions in a twofold helical conformation, in which each unit is rotated 180° relative to the previous unit.^[^ ^2^ ^]^ The glucan chains are stacked parallel to each other (in native cellulose type‐I) and held together via inter‐ and intrachain hydrogen bonding. In the literature there is no agreement on the number of glucan chains in the cellulose microfibril, and this probably varies with plant species. In cell wall molecular studies, a microfibril model of either 18, 24, or 36 glucan chains is adopted.^[^ ^4^, ^5^ ^]^ Hemicellulose is a carbohydrate with sugar units that differ between softwoods and hardwoods. Softwood hemicellulose consists of galactoglucomannans and arabinoglucoronoxylan and in hardwood of glucuronoxylan and glucomannan.^[^ ^2^ ^]^ Acetyl group decorations are present in galactoglucomannan and glucuronoxylan.^[^ ^6^ ^]^ Lignin is a noncrystalline macromolecular structure built up from phenyl groups. Both syringyl‐ and guaiacyl‐type (S/G) lignin units are present in hardwoods, and softwood lignin consists only of guaiacyl units.^[^ ^2^ ^]^

Cellulose is aggregated into structures called microfibrils (3–5 nm) that interact with hemicellulose and lignin to form a macrofibril (25–30 nm) (Figure 1a). The orientation of these macrofibrils varies within the layered structure of the wood cell wall (Figure 1a) which consists of four distinct layers (primary, S1, S2, and S3) with different thicknesses and chemical compositions. The anisotropic behavior, the mechanical performance and the shrinkage properties of wood are highly dependent on the microfibrillar orientation in the thick S2 layer.^[^ ^2^ ^]^

Lignin is the least abundant wood polymer component and is the cementing agent/matrix component of wood. Cellulose acts as the reinforcement in the composite structure of wood and hemicellulose is the bonding agent between lignin and cellulose (Figure 1a). Lignin concentration is correlated with higher stiffness and compressive strength in wood materials^[^ ^7^ ^]^ and cellulose is associated with its high tensile strength and stiffness.^[^ ^2^ ^]^

Just focusing on the mechanical properties alone, wood has a comparable Young's modulus (parallel to the grain of the wood; 9–16 GPa) to glass fiber reinforced plastic (GFRP; 7–45 GPa).^[^ ^8^ ^]^ Given its low density (pine, 0.52 kg m^−3^ or cork, 0.20–0.35 kg m^−3^),^[^ ^9^ ^]^ wood has very high specific mechanical properties, competing with other common materials such as metals, composites, and ceramics. These properties are best understood on an “Ashby‐plot” as illustrated in Figure 1b.^[^ ^10^ ^]^ The dotted and solid lines in Figure 1b represent points of constant *E/ρ* and *E/ρ* ^2^, respectively. The latter of these parameters is instructive since a high value represents a material, or structure, that has a high stability criterion, i.e., a high column that can withstand buckling at low density.^[^ ^10^ ^]^ As a construction material, wood possesses ideal properties for this purpose.

In addition, wood also has excellent optical properties, that can also be combined with high mechanical performance. Although nontransparent, wood can be made to be transparent through initial chemical bleaching, followed by refractive index matching with an appropriate material or substance.^[^ ^11^ ^]^ This approach was initially carried out by Fink, as a microscopical technique for analyzing wood morphology,^[^ ^11^ ^]^ but has recently been extended to look at a variety of functional materials, some of which have high stiffnesses and strengths.^[^ ^12^ ^]^

Wood is also accessible by other forms of radiation, most notably X‐rays,^[^ ^13^ ^]^ but also by optical and UV lasers giving typical excitations both for infrared and Raman absorption and scattering.^[^ ^14^ ^]^ In addition to these spectroscopies, confocal scanning laser and fluorescence microscopies (CSLM) can be used to image the structure of wood, particularly the location of components such as lignin.^[^ ^15^ ^]^ Spectroscopic approaches, while enabling a form of imaging based on chemical composition, also allow the locating of specific compositions of the wood.

The review now turns to describing in detail, and critically assessing, the development of NMR spectroscopies, X‐ray diffraction, Raman and infrared spectroscopies, confocal laser scanning microscopy (CLSM), and atomic force microscopies as tools for structural characterization of wood materials. Specific emphasis is on how the structure relates to the mechanical properties of the material, most importantly stiffness, strength, and fracture toughness.

## 2 Nuclear Magnetic Resonance (NMR) Characterization of the Molecular Structure of Wood

Over the years, various microscopic and spectroscopic studies have revealed the chemical composition and the structural arrangement of the cell wall. Emphasis has been placed on the orientation and dimensions of the cellulose microfibrils within both the primary and secondary walls. Despite the considerable understanding of the structural contribution and main functions of the cell wall components, there is limited knowledge on how these components are arranged and how they interact at the molecular level. Cell wall polymer conformations, crystallography, and spatial proximities at the level of sub‐nanometers are better detected with nuclear magnetic resonance (NMR) spectroscopy for both amorphous and crystalline structures.^[^ ^21^ ^]^ NMR studies are based on the electromagnetic signals emitted from polarized magnetic nuclear spins under a constant homogeneous magnetic field, *Β*_0_, after application of a second magnetic field, *B*_1_, at a radio frequency pulse. When *B*_1_ is applied at a resonant frequency both energy absorption (+1/2 nucleus shifts to −1/2) and emission (−1/2 nucleus shifts to +1/2) take place. When the rates of energy absorption and emission are equal the sample is at saturation point after which the nuclear spins return to a low energy state during a relaxation process. This process is characterized by a spin‐lattice (longitudinal) relaxation, yielding a time constant *T*_1_, and a spin–spin (transverse) relaxation, yielding a time constant *T*_2_. The variation in the resonant frequency of the nucleus due to the electron shielding effect (electron cloud surrounding the nucleus) is termed the “chemical shift” and is characteristic of the molecular structure and chemical bonds. The resonant frequency is related to the rotational frequency of the precessional motion of the nucleus’ magnetic moment under *B*_0_.^[^ ^22^ ^]^

Although NMR studies have developed our understanding of cell wall polymer interactions and molecular architecture, the degree to which these interactions affect the mechanical properties at a macroscale level is still not fully understood due to the complex hierarchical wood structure. Genetic modification of plants, such as *Arabidopsis*, is a common procedure adopted by biochemists in order to understand the cell wall polymers’ contribution to cell wall structure. *Arabidopsis* has been suggested as the plant model for hardwood species.^[^ ^23^, ^24^ ^]^ Despite modified *Arabidopsis* plants providing a simpler route to study the cell wall structure, it is often questioned if the findings can be directly related to woody dicots. However, since this is a standard model for the field, we use additional results of work on these plants as a review of the area.

NMR spectroscopy is conducted either in the solution or the solid state. Solution NMR uses either water (D_2_O) or other solvents (e.g., DMSO‐*d*_6_ to identify lignin–carbohydrate linkages combined with chromium acetylacetonate to provide complete relaxation of all nuclei). High‐resolution spectra are derived from solution NMR attributed to the higher molecular motion resulting in narrow peaks compared with solid‐state NMR. However, solid‐state NMR studies are more representative of the native structure of wood and are suitable for wood polymer components such as cellulose.^[^ ^25^ ^]^ The resolution in solid state NMR spectroscopy increases with the use of ^13^C cross‐polarization magic angle spinning (CPMAS) spectra where dipolar couplings and chemical shift anisotropy effects are eliminated. Therefore, the chemical shifts of equivalent carbons with different magnetic fields can be differentiated.^[^ ^25^ ^]^ Cross‐polarization enhances the signal sensitivity of rigid molecular structures whereas direct polarization can be used for less rigid components and quantitative spectra.^[^ ^26^ ^]^ Signal overlapping problems have been reported in 1D CP‐MAS NMR studies, but the use of periodically varying pulses at different time periods with multidimensional NMR studies can yield multiple information about the molecular structure and proximities. Double quantum correlation with the refocused INADEQUATE pulse sequence has been adopted for the detection of through bond interactions in cell walls.^[^ ^27^ ^]^ The method relies on J‐couplings between nuclei where C–C homonuclear dipole–dipole couplings are removed by the MAS probe and through‐space interactions are suppressed.^[^ ^28^ ^]^ 2D Heteronuclear single quantum (HSQC) NMR spectroscopy has been used extensively to identify lignin–carbohydrate linkages^[^ ^29^, ^30^ ^]^ allowing peaks in the ^1^H–^13^C system to be detected. ^13^C–^13^C proton driven spin diffusion (PDSD) NMR experiments have been adopted for through space intermolecular bond interactions.^[^ ^4^, ^24^, ^26^ ^]^ A longer mixing time in the pulse sequence is suitable to detect higher spatial proximities between nuclei up to a maximum distance of 5–10 Å.^[^ ^24^ ^]^ The use of a cryogenic probe can result in a much higher resolution with a threefold signal enhancement^[^ ^31^ ^]^ and it has been successfully applied to resolve overlapping signals of lignin carbohydrate complex γ‐ester linkages and lignin acetyl γ‐esters.^[^ ^29^ ^]^

It is known, from CP‐MAS NMR studies, that native cellulose in plants exists in two forms: the single‐chain triclinic Iα crystal and the two‐chain monoclinic Iβ crystal.^[^ ^32^ ^]^ Although it has been reported that crystalline forms like Iβ dominate in wood,^[^ ^33^ ^]^ NMR studies have shown multiple signals for cellulose carbons exhibiting a more heterogeneous structure. This heterogeneous structure is present not only along the chains but also across the section of the cellulose microfibril^[^ ^21^ ^]^ as indicated by the spatial proximities of different glucosyl environments. It has been suggested that two main cellulose domains exist within the cellulose microfibril exhibiting distinctive chemical shifts at carbons 4 and 6 of the glucose unit: cellulose domain 1 is associated with a crystalline form at the interior of the cellulose microfibril and cellulose domain 2 related to a less ordered form at the surface chains of the cellulose microfibril.^[^ ^21^, ^24^, ^34^ ^]^ CP‐PDSD NMR experiments on spruce wood suggest that cellulose domains 1 and 2 constitute 42% and 58% of the cellulose microfibril respectively.^[^ ^24^ ^]^ Revealing the cellulose structure was difficult in the past due to overlapping of the cellulose chemical shifts with other polysaccharides, but these shifts are better resolved using 2D solid‐state NMR.^[^ ^4^ ^]^ Agarwal et al.^[^ ^35^ ^]^ proposed a provisional cellulose microfibril model with cellulose chain regions divided into water accessible (noncrystalline) and inaccessible areas according to data from Raman spectroscopy (either in the dry state or in the presence of H_2_O and D_2_O) and WAXS studies of control and treated softwood and hardwood samples. To a greater extent, a redefinition of the surface and interior cellulose domains from the C4 and C6 peaks in the ^13^C NMR spectrum was proposed. The 89 and 84 ppm peaks can be attributed to water inaccessible and accessible C4s and the 66 and 64 ppm peaks, associated with the trans–gauche (tg) and gauche–trans (gt) conformation, respectively, can be related to C6 water inaccessible and accessible areas. The noncrystalline forms of cellulose were attributed to the variety of hydroxy methyl conformations with gt conformation disturbing stable interchain hydrogen bonds. Small amounts of gauche–gauche (gg) conformations were also detected. Multiple glucose environments in spruce secondary cell walls were also reported with the assignment of three distinctive chemical shifts in both cellulose domains 1 and 2.^[^ ^24^ ^]^ Several degrees of crystallinity have been reported in both softwood and hardwood attributed to the different pretreatment regimes and signal overlapping.^[^ ^25^ ^]^ A 3% lower cellulose crystallinity has been reported in hardwoods compared with softwoods^[^ ^36^ ^]^ and lower crystallinity has been measured in extracted cellulose from *Eucalyptus* heartwood than for sapwood.^[^ ^37^ ^]^

**Figure 2:**
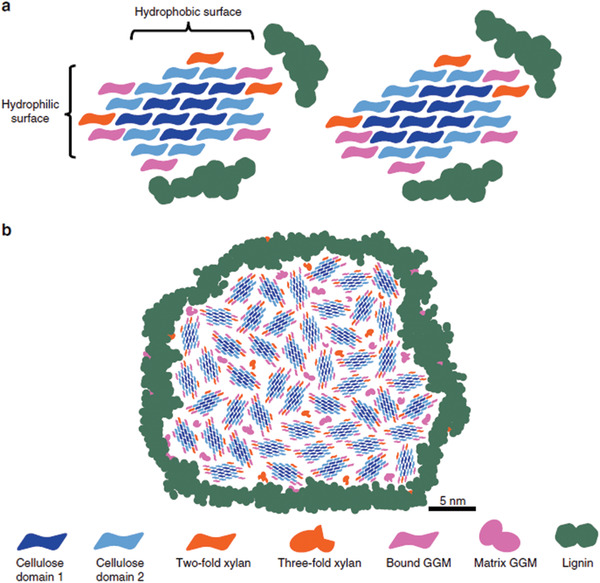
Proposed model of softwood molecular architecture: a) microfibril cellulose structures and hemicellulose–cellulose interactions and b) macrofibril structure. a,b) Reproduced under the terms of the CC‐BY Creative Commons Attribution 4.0 International license (https://creativecommons.org/licenses(by/4.0).^[^ ^24^ ^]^ Copyright 2019, The Authors, published by Springer Nature.

The information derived from NMR studies has increased our understanding of the cell wall molecular architecture and several cell wall models have been proposed based on the proximities of the different wood polymer components. Terrett et al.^[^ ^24^ ^]^ conducted multidimensional solid‐state (MAS) NMR studies on Norway spruce native samples and suggested that xylan forms a flattened twofold screw conformation along the hydrophilic surfaces of the cellulose microfibrils enabling a stable interaction with cellulose. The same xylan–cellulose interactions were also proposed for *Arabidopsis* secondary cell walls.^[^ ^38^ ^]^ Close proximities between cellulose domain 1 and both galactoglucomannan (GGM) and xylan were observed suggesting that xylan is bound to cellulose domain 2 via hydrogen bonding. This modifies its crystallinity structure closer to cellulose domain 1 as a result of the change in the cross‐peak signals from the CP‐PDSD NMR experiments. Other potential factors affecting the signals could be a “bundle” effect between microfibrils or different orientations of the hydroxymethyl groups in the surface chains.^[^ ^26^ ^]^ The possibility of hemicellulose intercalation into cellulose domain 1 was excluded.^[^ ^24^ ^]^ This contradicts the proposed primary cell wall model for *Arabidopsis thaliana* where xyloglucan is entrapped into the cellulose microfibril contributing to a more rigid structure.^[^ ^27^ ^]^ However, this structural arrangement might be affected by the prevalent pectin‐cellulose and pectin‐xyloglucan interactions in the primary cell wall of *A. thaliana* as observed with 3D MAS NMR. GGM and xylan are in close proximity (within 5–10 Å) and bound to the same or adjacent cellulose microfibril^[^ ^24^ ^]^ (see **Figure** **2**a) and therefore there is no space for lignin to interact with cellulose as proposed in other cell wall models.^[^ ^39^, ^40^ ^]^ However, NMR studies showed that some lignin is associated with both xylan and GGM and cellulose domain 2.^[^ ^24^ ^]^ These interactions were suggested to occur between macrofibrils where lignin is expected to be aggregated (see Figure 2b). Perez^[^ ^27^ ^]^ suggested that lignin is bound favorably to threefold xylan conformations and that xylan and lignin interact with sub‐nanometer electrostatic interactions rather than through covalent bonds. Simmons et al.^[^ ^26^ ^]^ argued that there is an interdependence between the xylan folding conformation and cellulose by comparing wild type with cellulose deficient *Arapidopsis* stems. In the latter, the threefold xylan conformation was dominant. Small amounts of threefold xylan conformations have been detected in both wild type *Arabidopsis* plants^[^ ^26^ ^]^ and spruce wood^[^ ^24^ ^]^ suggesting that threefold xylan exists as a matrix component between microfibrils either in an unbound form or bound to cellulose in an incompatible way.^[^ ^24^ ^]^ Busse‐Wicher et al.^[^ ^41^ ^]^ showed, with the aid of molecular dynamic simulations supported by data from NMR studies, that xylan in *A. thaliana* can bind in various ways with cellulose, including interaction with the hydrophobic surfaces, helical wrapping of the cellulose microfibril to bind with the hydrophilic surfaces, and the forming of loops along the hydrophilic surfaces.

**Figure 3:**
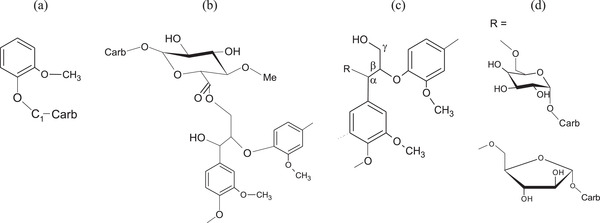
Main lignin–carbohydrate linkages: a) phenyl glycoside, b) γ‐ester, and c) benzyl ether. Adapted with permission.^[^ ^44^ ^]^ Copyright 2007, De Gruyter.

NMR studies have shed light on the types of lignin–carbohydrate (LC) interactions that have been postulated to be α‐ester, α‐ether, and phenyl glycoside bonds (see **Figure** **3**) based on indirect methods such as chemical degradation techniques and model compounds. However, due to the low amount of the LC bonds (3 per 100 C_9_^[^ ^42^ ^]^) in wood, fractionation procedures, e.g., isolation of milled wood lignin and extraction of LC fragments enriched in carbohydrates, are necessary to enhance NMR signals. Enhancement of signals in LC fragments can also be achieved with C‐labeling methods.^[^ ^43^ ^]^ NMR studies have shown the absence of α‐ester LC bonds.^[^ ^44^, ^45^ ^]^ Signals suggesting the presence of phenyl glycoside, α‐ether, and γ‐ester LC bonds have been detected in both softwood^[^ ^44^ ^]^ and hardwood species.^[^ ^46^ ^]^ Several phenyl glycoside signals have been reported,^[^ ^45^, ^46^ ^]^ attributed to different sugar units bound to hydroxyl groups in lignin. Du et al.^[^ ^45^ ^]^ contended that mannose and galactose are the main polysaccharide units involved in the LC linkages based on the characterization of enzymatically hydrolyzed LCCs from spruce wood with ion exchange chromatography. Yet, the relative composition of sugar units in LCCs varies between acetic acid and enzymatic preparations.^[^ ^29^ ^]^ In most NMR studies, the identification of LC linkages relies on chemical shifts derived from model compounds.^[^ ^44^, ^47^ ^]^ Nishimura et al.^[^ ^48^ ^]^ managed to identify α‐ether LC bonds with connectivity analysis and complete assignment of all signals within a lignin–carbohydrate complex (LCC) by conducting long range 3D‐NMR studies.

Consistent guidelines in specimen preparation are needed in order to enable the research community to compare experimental findings from test regimes that simulate better the native structure of plants. Chemical and biochemical enzymatic methods have been used to infer the cell wall composition and the number and spacing of substrate substitutions. However, these methods might lead to incomplete digestion of the cell wall polymer components due to interactions of the enzymes with backbone decorations,^[^ ^41^ ^]^ or due to steric limitations,^[^ ^49^ ^]^ and they cannot provide thorough understanding of the type of bonds between cell wall polymer components.^[^ ^44^ ^]^ Despite these experimental procedures being tedious due to the required pretreatment, e.g., delignification, they can complement and verify experimental findings from other cell wall imaging techniques. Extraction of cell wall polymers is usually adopted due to enzymatic inaccessibility or due to spectral congestion, e.g., signals of mobile cellulose domains overlapping with hemicellulose signals.^[^ ^50^ ^]^ Chemical extractions can modify the molecular structure of cell wall components leading to erroneous results and uncertainties in our understanding of the cell wall molecular architecture. Extraction of cell wall components using alkaline treatment can result in a decreased cellulose crystallinity and re‐arrangement of the noncellulosic polysaccharides.^[^ ^51^ ^]^ However, a higher degree of cellulose crystallinity has been reported by comparing the CP‐MAS spectra of isolated cellulose with original wood samples.^[^ ^50^ ^]^ Ball milling of wood and fractionation of LCCs is necessary for better identification of the LC bonds due to their low frequency in wood cell walls in combination with signals overlapping in certain low‐sensitivity NMR analyses.^[^ ^29^, ^48^ ^]^ Additional difficulties lie in the high molecular weight of some LCCs enriched with polysaccharide chains affecting the spin–spin relaxation time (*T*_2_).^[^ ^52^ ^]^ Most common LCC preparations found in the literature are acetic acid^[^ ^29^, ^44^ ^]^ and enzymatic hydrolysis.^[^ ^45^, ^48^ ^]^ Differences in specimen preparation can modify the LCC molecular structure often leading to misinterpretation of the signals in NMR studies. Lignin degradation and cleavage of β‐Ο‐4′ linkages take place during ball milling procedures and LCC linkages can be affected during acetic acid preparations.^[^ ^46^ ^]^ Balkashin et al.^[^ ^44^ ^]^ contended that deacetylation of hemicellulose in alkaline treatments of LC samples can result in misidentification of α‐ester LC bonds. Alkaline treatment results in suppression of the γ‐ester bond signals.^[^ ^29^, ^45^ ^]^ Enzymatic hydrolysis with cellulolytic enzymes can cleave phenyl–glycoside bonds.^[^ ^29^, ^45^ ^]^ Acetic acid (Ac‐OH) and cellulolytic enzyme lignin (CEL) preparations were compared in both hardwood and softwood samples.^[^ ^29^ ^]^ Acetic acid preparation was more effective in pine wood with a higher yield in LCCs. However, acetic acid preparation seemed to severely degrade lignin, based on the lower amounts of β‐O‐4, β‐β, and β‐5 structures potentially affecting the LCC linkages. Benzyl‐ether linkages were better identified in CEL preparations for both hard‐ and softwood, and acetic acid softwood LCCs were suitable for phenyl‐glycoside and ester LCC linkages. There is no single LCC preparation suitable to depict both the lignin structure and all LC interactions.^[^ ^29^ ^]^ In conclusion, it is questionable how to extrapolate and relate the findings from LCCs and extracted cell wall polymers to the real molecular structure of the wood in vivo.

In solid state NMR studies native wood specimens are tested with various moisture contents. There is no specific preconditioning regime for the specimen preparation. Yet, moisture content variations in samples can lead to different findings for the cell wall molecular architecture.^[^ ^34^ ^]^ The presence of water can increase the microfibril dimension and the monoclinic angle as suggested in^[^ ^50^ ^]^ based on WAXS data, affecting the cellulose resonances,^[^ ^34^ ^]^ and changing the xylan conformation to a threefold screw axis.^[^ ^26^ ^]^ Moreover, due to higher molecular motion the NMR signals can be affected.

## 3 X‐ray Diffraction Studies on Wood Microfibrillar Features and Mechanics

**Figure 4:**
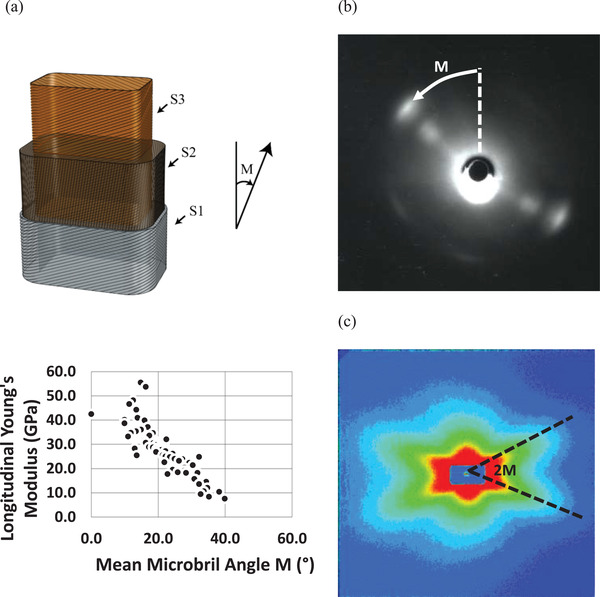
a) Schematic of a wood cell tracheid showing the microfibril angle (*M*) in the S2 layer top). The relationship between the longitudinal Young's modulus of *Pinus radiata* and M bottom). Data are reproduced from Cave^[^ ^54^ ^]^ with units converted to SI; typical diffraction patterns from wood cell walls. b) Wide‐angle X‐ray diffraction pattern and c) small‐angle X‐ray diffraction pattern showing how to determine *M*. b,c) Reproduced with permission.^[^ ^58^ ^]^ Copyright 2001, Springer Nature.

The scattering of X‐rays from wood is complex, but nevertheless rich in information about the various structural aspects of its makeup. It has been known for some time that the scattering of both small‐ and wide‐angle X‐rays from wood cell walls yields information on the pronounced microfibril angle within the S2 layer of the wood cell walls.^[^ ^53^ ^]^ The relationship between the Young's modulus of the woody tissue, and the microfibril angle (M) has been well established (**Figure** **4**a).^[^ ^54^ ^]^ Typically, the scattering of wood, at small and wide angles, yields patterns that are distinguishable in features. Wide angle patterns yield diffraction spots that are at an angle to the meridian of M (Figure 4b), whereas small angle patterns (Figure 4c) are typified by distinct cruciform streaks that are separated by an angle 2M. More details on this analysis can be found in the work by Entwistle et al.^[^ ^55^ ^]^

**Figure 5:**
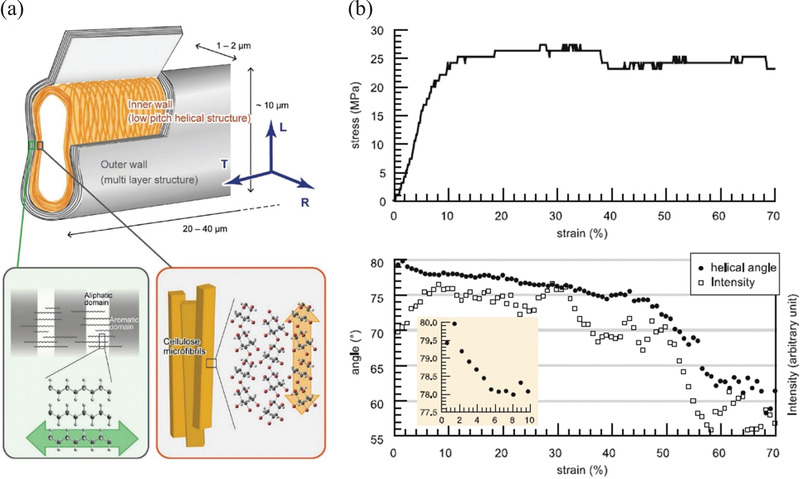
a) Schematic of the cell wall structure for fibers found in cherry bark (*C. sargentii*) showing (top) the flattened geometry of the fibers indicating dimensions and longitudinal (L), transverse (T), and radial (R) directions and (bottom) the compositions of the outer layer of the fibers of aliphatic/aromatic lipids and the inner layer of helically wound cellulose microfibrils. b) A typical stress–strain curve of cherry bark top) and the change in microfibril helical angle and the intensity of the scattering peaks used to derive this quantity bottom). The inset shows a close‐up view of the change in helical angle in the elastic regime. a,b) Reproduced with permission.^[^ ^57^ ^]^ Copyright 2018, Wiley‐VCH.

The relationship between the deformation of wood, and a commensurate change in the microfibril angle, as derived from X‐ray diffraction patterns, has been reported by Keckes et al.^[^ ^56^ ^]^ They showed quite clearly that there is a monotonic change in the fibril angle (toward a smaller value), as wood tracheids are deformed in tension, and furthermore that this change is reversible, leading to elasticity based on a “Velcro” effect within the cell walls.^[^ ^56^ ^]^ Although not a bulk wood sample, exceptionally high strains to failure have been observed for cherry bark (*Cerasus sargentii)* by Kobayashi et al.^[^ ^57^ ^]^ Fibril angles of >70°, indicative of a helical structure to the cellulose, have been observed for this material (**Figure** **5**a). These helical microfibrils are also lignified. The outer walls are however found to mainly contain a lipid polymer—suberin. This composite structure leads to the high strains observed. Kobayashi et al.^[^ ^57^ ^]^ used synchrotron X‐ray diffraction to follow the change in the fibril angle with tensile deformation, providing a correlation with mechanical properties (Figure 5b). It was noted that these stress–strain curves were reminiscent of a thermoplastic polymer, leading to a high “toughness” (or work of fracture; ≈50 MJ m^−3^).

**Figure 6:**
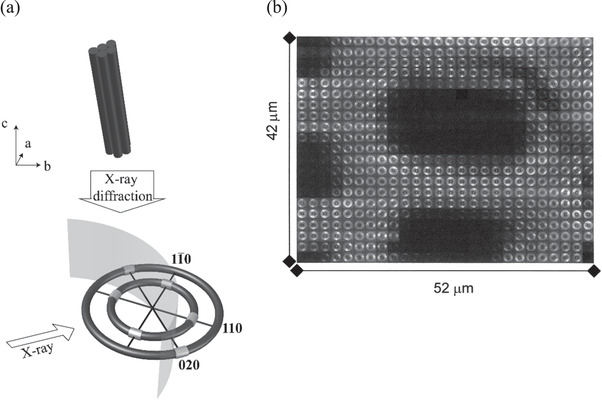
a) Schematic of a typical X‐ray diffraction pattern from the end section of a wood cell wall indicating the approach to determine the microfibril angle; b) mesh scan over a complete wood cell in cross section with parts of neighboring cells, pixel size: 2 × 2 mm. Dark regions correspond to lumina, bright regions showing a scattering signal correspond to cell walls. Each pixel corresponds to an individual diffraction pattern from which the microfibril angle can be derived. a,b) Adapted with permission.^[^ ^59^ ^]^ Copyright 1999, International Union of Crystallography (Reproduced with permission of the International Union of Crystallography; https://doi.org/10.1107/S0021889899010961).

Improvements in the optics and acquisition times, using synchrotron radiation, has additionally enabled spatial resolution of the fibril angles in wood specimens. Lichtenegger et al.^[^ ^59^ ^]^ demonstrated that having the beam incident along the longitudinal axis of wood cell walls allowed imaging of the fibril angles in cell walls, enabled by the unique scattering patterns obtained due to an asymmetry effect in the 2D scattering pattern (**Figure** **6**a). Such imaging allowed a spatial resolution of ≈2 µm, and with scan times of 16 s, enabling maps that are comparable to some optical microscopy images, but with detailed and quantitative structural detail (Figure 6b).

Most structural imaging techniques rely on 2D visualizations of the wood surface topology. Several 3D imaging methods have been developed and used as probes of plant materials, but usually at the expense of resolution. Optical projection microscopy (OPT) with either UV or visible light at a spatial resolution of 5 × 5 × 5 µm has been used to reveal the internal structure of *Arabidopsis* siliques.^[^ ^60^ ^]^ However, pretreatment of the samples with organic solvents is required to attain higher resolution images with transmission OPT. Therefore, this seems to be more suitable for growth dynamics studies of roots and seedlings due to their inherent semitransparency. Wood's unique cellular structure lends itself to other X‐ray imaging techniques, such as tomographical imaging. This technique has until recent times been reserved for more conventional materials. X‐ray computed tomography is an advanced noninvasive 3D imaging technique with limited sample preparation providing spatial resolution down to a sub‐micrometer level (0.7 × 0.7 × 0.7 µm).^[^ ^61^ ^]^ While the imaging in 2D for conventional X‐rays is rich in detail, tomographical imaging allows 3D structural information, with the addition of a temporal dimension to spatial mapping. Therefore, 3D reconstructions of a series of 2D projections can be built with the aid of advanced image software. Detailed descriptions of how tomographical imaging works is covered elsewhere.^[^ ^62^ ^]^ Higher resolution and faster scanning times can be achieved with synchrotron radiation.^[^ ^63^ ^]^ Large‐scale investments in synchrotron radiation facilities (e.g., ESRF in France, Diamond Light Source in the UK, SPring‐8 in Japan) have enabled microfocus and sub‐micrometer focus beams to be used for the characterization of wood. This ability to map at a smaller length scale, and with faster acquisition times brings together spatial–temporal scales for looking at real‐time deformation. For X‐ray CT measurements this enables much more rapid constructions and images of wood to be taken. One of the key challenges of X‐ray tomography is the trade‐off between data storage, scanning time, and resolution. X‐ray CT scan images are discretized into subvolumes (voxels). Increasing the resolution of the voxel will result in a cubic growth in data.^[^ ^63^ ^]^ Multiple scans of increment cores (60 cm (diameter) × 100 cm (height)) for automated tree growth ring studies have been tried with X‐ray CT scanning but at the expense of resolution.^[^ ^63^ ^]^ More complicated scanning set ups with two X‐ray tubes and detectors can lead to a higher resolution (sub‐micrometer level) but usually at the expense of experimental measurement times.^[^ ^63^ ^]^ X‐ray computed tomography has been used in wood research for both qualitative and quantitative description of the anatomical features in wood species, e.g., wood porosity, cell wall thickness and vessel structural network, density measurements, time‐dependent studies of water diffusion processes, and chemical treatment penetrations but also for mechanical testing. More details on the application of X‐ray tomography in wood studies can be found in Van den Bulcke et al.^[^ ^64^ ^]^ Here, we highlight specific examples of its applicability to the understanding of mechanical properties.

The potential of temporal synchrotron‐based tomography to make dynamic measurements of the mechanical properties of wood has been realized through studies that have followed the change in structure during compression.^[^ ^65^, ^66^, ^67^ ^]^ These studies have clarified mechanisms of deformation in wood‐based materials, including demonstrating that the external porosity (external to the fibers) plays the most critical role in the transverse compression of low density fiberboard.^[^ ^65^ ^]^ The fibers themselves were not found to deform significantly in compression, which was only revealed from the in situ imaging of the structure during deformation.^[^ ^65^ ^]^

Other work of note has been the ability to track the ingress of adhesives into bonded wood joints.^[^ ^67^ ^]^ This work has significance because there is an increasing interest in using joining wood veneers, flakes, strands, and particles in construction materials, an approach that can displace conventional materials like concrete and mitigate CO_2_ emissions. The approach here was to use tomographical scans to build up a realistic structure of wood, which was subsequently used in a flow model of adhesive penetration.^[^ ^67^ ^]^ These models can then be used to better understand the mechanical performance of composites of wood and adhesives for emerging areas such as cross‐laminated materials.^[^ ^68^ ^]^ They can also be informative for chemical impregnation treatments to increase the durability performance of engineered wood but also for the drying kinetics within wood to increase the kiln drying efficiency that is responsible for up to 90% of the total manufacturing energy in construction timber products.^[^ ^69^ ^]^

## 4 Raman and Infrared and Imaging of Wood

Spectroscopic techniques have spatial resolution limits depending on the wavelength of the light. The maximum spatial resolution of confocal Raman spectroscopy is in the range 250–300 nm. Given that most polymers, including cellulose (which is the main composition of wood), have very large band gaps, they yield relatively low intensity Raman spectra compared to other materials. For this reason, longer acquisition times have been typically required, limiting the possibilities for real‐time studies of the mechanical deformation of wood. It is well known that the deformation of polymeric materials, in particular fibers, results in a downshift in the position of Raman bands.^[^ ^70^ ^]^ The specific mechanism behind this downshift is covered elsewhere^[^ ^70^ ^]^ but it relates to a direct molecular deformation of the polymer backbone chains. Early studies on the deformation of wood showing that is was possible to observe these downshifts,^[^ ^71^ ^]^ but with limited resolution in the data. Later studies, on thinner sections of wood, reported clear shifts in a Raman band located at ≈1096 cm^−1^ which is associated with the stretching modes of C—O and C—O—C groups along the cellulose backbone.^[^ ^72^ ^]^ No shifts were observed for the bands associated with lignin, which confirms that this material acts as a matrix to transfer stress, as for conventional composite materials.

Where Raman spectroscopy has made great advances in our understanding of wood has been through imaging. Confocal Raman spectroscopy is capable of imaging structures using chemical mapping, which can be obtained in two and possibly even three dimensions. Pioneering work by Gierlinger et al.^[^ ^73^ ^]^ showed that it was possible to spatially map the cell walls of poplar wood using unique spectral information emanating from cellulose, lignin and other carbohydrates. Raman spectroscopy proved useful in this respect since scattering from lignin, located in the spectral region 1550–1640 cm^−1^, enabled its unique identification, and where this compound is most dominantly located in the cell walls.^[^ ^73^ ^]^ Higher lignin concentrations were found in the cell corners (CC) and compound middle lamella (CML) regions of the cell walls. Additional analysis of other bands, for example those emanating from both lignin and carbohydrates other than cellulose revealed that the S2 layer of the cell walls are richer in the latter. Finally, a Raman band located at ≈1096 cm^−1^ was used to obtain orientation of the fibril angle, showing variation depending on which layer of the wood cell wall is imaged (S1 to S3). This same approach has been extended to obtain high precision, and quantitative information of the microfibril angle from different parts of wood cell walls.^[^ ^74^ ^]^

Infrared spectroscopy has been used to characterize woody tissues, most notably for intact trees and for the prediction of mechanical properties. In early work it was shown that features within near‐infrared (NIR) spectra could be correlated with mechanical properties,^[^ ^75^ ^]^ and underlying structural characteristics such as the microfibril angle.^[^ ^76^ ^]^ Statistical methods, such as principal component analysis (PCA)^[^ ^77^ ^]^ and projection to latent structures (PLS),^[^ ^78^ ^]^ were used to group and correlate characteristics, with correlation coefficients often exceeding 0.8.^[^ ^75^, ^76^ ^]^ The concept of using NIR enabled the use of hand‐held spectrometers in the forest to determine ultimate and important properties of timber, e.g., mechanical properties.^[^ ^76^ ^]^ Dynamic Fourier transform infrared (FTIR) spectroscopy has also been used to better understand the mechanical properties of woody materials, in particular interactions between different components (e.g., with lignin, hemicellulose) and under moist conditions. Initially work focused on wood pulps, and the prediction of Iα and Iβ compositions,^[^ ^79^ ^]^ but has since extended also to understanding mechano‐sorptive creep in cellulosic materials, such as wood, but using paper as an analogue material.^[^ ^80^ ^]^ Here it was shown that during creep under moist conditions, cellulosic materials expose more –OH groups to deuterium exchange, which suggests a sliding motion of the cellulosic chains during deformation.^[^ ^80^ ^]^ The orientation of other cell wall components, such as xylans, lignin and glucomannan, has also been shown to most closely follow that of the cellulose in compression wood, compared to “normal wood.”^[^ ^81^ ^]^ WAXS was used to measure the microfibril angle in both compression and normal wood sections, and these measurements were correlated with polarized IR measurements of the relative intensities of bands specific to components to a reference.^[^ ^81^ ^]^ Both glucomannan and xylan were found to be closely associated with the cellulose in both compression and normal wood, with the former more so than the latter, suggesting it is mostly involved in cellulose structural organization.^[^ ^81^ ^]^ Lignin was found to be most closely oriented with respect to cellulose in compression wood, compared to normal wood,^[^ ^81^ ^]^ which gave more detail to earlier studies showing a planar orientation of this material.^[^ ^82^ ^]^

One major advantage of infrared over Raman spectroscopy is that it is less prone to the obscuring of signals due to fluorescence. This is due to the fact that Raman scattering is particularly weak, being inversely proportional to the fourth power of the wavelength of the visible light used, which is also true for the more dominant elastic scattering.^[^ ^83^ ^]^ Given that plant materials are known to fluoresce, it is often difficult to obtain clear Raman spectra from them without obscuration from fluorescent chromophores within the structure. This was an issue for the early Raman studies on plant materials in the 1980s, where fluorescence was overcome using a variety of methods.^[^ ^84^ ^]^ One other major difference between these two types of spectroscopy is their spatial resolution. Since the spatial resolution (*r*) of a microscope is related to the numerical aperture (NA) of the lens used, and Abbés diffraction limit (*r* = 0.61λ/NA). It is clear then that the best spatial resolutions are achieved using UV (low λ) lasers, with NIR and IR lasers (high λ) giving the lowest resolution; this means that IR spectroscopy is somewhat more limited spatially (5–20 µm laterally) than Raman spectroscopy (0.3–2 µm laterally). Immersion optics, which are often used in Raman spectroscopy to enhance the NA of a lens system (to ≈1.4), are not available to IR spectroscopy due to absorption by the oil.^[^ ^83^, ^85^ ^]^ Some increase in spatial resolution is afforded to IR spectroscopy through the use of attenuated total reflectance (ATR) and the use of a crystal that impinges on the sample's surface, thereby increasing the NA.^[^ ^83^, ^86^ ^]^ Other issues with infrared and Raman spectroscopies are the need for sample preparation. Since infrared relies on the transmission of the incoming radiation, with the exception of ATR, thin sections are required, and also flat surfaces to those samples. While sectioning is not necessarily required for all Raman measurements, it is usually preferred for imaging purposes.

## 5 Electron Microscopy (EM) of Wood Cell Wall Ultrastructure

Over the last 50 years, EM has been a technique that has been used to study the cell wall ultrastructure.^[^ ^87^ ^]^ EM is carried out in vacuo and relies on detecting signals either from the emitted electrons (backscattered primary, secondary and X‐rays) from the sample (scanning electron microscopy, SEM) or transmitted electrons through the sample (transmission electron microscopy, TEM). Therefore, SEM is more suitable to study the surface morphology of wood and TEM can reveal more information on surface irregularities and internal anatomy.^[^ ^87^, ^88^ ^]^ SEM resolution can be limited to 1 nm whereas spatial resolution up to 0.1 nm can be attained with TEM.^[^ ^88^ ^]^ At long scanning times SEM provides images with higher depth of field enabling 3D visualization of wood. However, wood samples are more susceptible to beam electron radiation damage at long scanning exposure times. In SEM nonconducting materials like wood are coated with a thin layer of heavy metal such as gold or platinum. The thickness of the coating can play a significant role on the resolution of the images with thin coatings, e.g., 3 nm yielding more information on the surface morphology of the wood cell wall.^[^ ^23^, ^89^ ^]^ With SEM the specimens can be observed either in their native (e.g., cryo‐SEM) or in a dried state. Several drying methods exist, such as freeze drying or air drying, but they can lead to cross shrinkage, distortion and collapse of thin cell walls.^[^ ^87^ ^]^ On the other hand, cryofixation and deep etching avoids cell wall damage with the formation of regular shaped ice crystals.^[^ ^87^ ^]^ Extensive literature reviews on the specimen preparation procedures and different electron microscopy methods (transmission electron microscopy, TEM; scanning transmission electron microscopy, STEM, and SEM) for the ultrastructure of wood can be found elsewhere.^[^ ^87^, ^89^ ^]^

Environmental scanning electron microscopy (ESEM) has been used in combination with mechanical tensile testing,^[^ ^90^ ^]^ but with limitations in the applied strain rates. A slow electron beam scan rate (2.3 frame s^−1^) enabled image noise minimization and was judged suitable to avoid electron beam‐related radiation damage. Strain distribution profiles on the surface of flattened latewood fibers in black spruce (*Picea mariana*) could be derived by tracking surface texture patterns with increasing deformation and applying digital image correlation on SEM photos. However, static images were captured at specific extension values during testing that could have led to additional creep effects and nonuniform strain profiles. Another study that combines in situ electron microscopy studies with mechanical testing is the investigation of crack propagation in the S2 layer and S1/S2 interface of cell walls in pine latewood.^[^ ^91^ ^]^ Mode I fracture experiments with a modified double cantilever beam geometry were carried out revealing rapid crack development (brittle type) along the microfibril orientation toward the S1/S2 layer. At the S1/S2 interface a “zig‐zag” crack development, attributed to the out of plane orientation of the microfibrils and the different angles at the S1/S2 transitional layer, increased the fracture toughness. This complex local microfibrillar architecture at the S1/S2 led to crack arrest, crack tip blunting due to viscoelastic deformation and crack bridge formation. Reza et al.^[^ ^92^ ^]^ showed with TEM images that past proposed cell wall models with concentric planar helical arrangements of microfibrils are not valid. TEM images of radial, tangential and transverse sections of Norway spruce wood showed that the microfibrils in S1 and S2 layer exhibit an out of plane orientation toward the lumen. Changes in the microfibrillar orientation in the S2 layer were visible even at low magnification but the S2/S3 transition layer could not be clearly distinguished and a microfibrillar entanglement was proposed. The S1/S2 layer was clearly distinguished due to a sudden change in the microfibrillar orientation.

A recent cryo‐SEM study^[^ ^23^ ^]^ of the secondary cell wall structure of both softwood (spruce and Ginkgo) and hardwood (poplar) samples showed that softwood exhibits a larger macrofibril diameter (≈34 nm) compared with hardwood species (≈18 nm), which was attributed primarily to the presence of galactoglucomannan in the former. The findings were based on a statistical analysis of 150 macrofibril size measurements. In the same study the contribution of cellulose, xylan, lignin, and xylan–cellulose interactions in the microfibril aggregate arrangement was elucidated with genetically modified *Arabidopsis* stems.^[^ ^23^ ^]^ Xylan and lignin deficient stems showed up to 30% and 15% reduction in macrofibril diameter respectively proportionally to the relevant decrease in the cell wall polymer content. Cellulose deficient stems had no visible fibers, and modified stems with reduced acetylation decorations and lack of an even acetylation pattern exhibited an approximately 25% decrease in the macrofibril size.

EM studies are rich in information regarding the microstructural arrangement of cell walls and microfibrils, surface textural configurations and impregnation of polymers in the cell wall and can complement existing imaging spectroscopic studies.

## 6 Confocal Laser Scanning Microscopy Imaging of Wood Structure and Interactions

Confocal laser scanning microscopy (CLSM), commonly shortened to confocal microscopy, is a technique that has been used comprehensively in the study of natural materials, most notably cellulosic. The technique was first patented by Minsky,^[^ ^93^ ^]^ and the principal subjects of his confocal microscope in 1955^[^ ^93^ ^]^ and the first confocal laser scanning microscopy (CLSM) studies in the late 1980s^[^ ^94^ ^]^ were biological human tissues and cells. This exemplified the practical relevance of CLSM in the study of wood tissues and cells in the early 1990s.^[^ ^95^, ^96^, ^97^, ^98^ ^]^

In conventional wide‐field optical microscopy, light (whether scattering, transmitting, reflecting or fluorescing) from out‐of‐focus planes of the specimen produces a degraded, hazy image of the specimen in the focal plane.^[^ ^99^, ^100^ ^]^ In his memoir on inventing the confocal microscope, Minsky writes that “the way to avoid all that out‐of‐focus scattered light was to never allow any unnecessary light to enter in the first place.”^[^ ^93^ ^]^ In the confocal microscope, this was achieved by pinholes placed at the light source and the detector, which were both “confocal” to a single point in the focal plane of the sample.^[^ ^99^, ^100^, ^101^, ^102^ ^]^

Using this spatial filtering technique, and by raster scanning point‐by‐point through a region of interest, optical sections or slices of the sample at high lateral *x–y* resolution (≈0.25‐0.40 µm) can be produced for a very low depth of field (<1 µm).^[^ ^98^, ^103^ ^]^ Confocal scanning microscopy remained largely unexploited until the late 1980s when the use of laser beams as the light source offered the powerful illumination needed to obtain fluorescence images from deep within the sample. Using a laser beam also enabled faster scanning and the acquisition of higher quality images with minimal sample preparation in comparison to epifluorescence microscopy.^[^ ^93^, ^100^, ^104^ ^]^ Moving the acquisition focal plane deeper into the object in well‐defined *z*‐steps of size <200 nm^[^ ^98^, ^105^ ^]^ to >10 µm,^[^ ^103^ ^]^ though typically around 0.5–1.5 µm,^[^ ^98^, ^106^ ^]^ allows the collection of optical sections even 100 µm below the sample wood surface.^[^ ^103^, ^105^, ^107^ ^]^ The thickness of the optical section is determined by the optical arrangement,^[^ ^98^ ^]^ namely, size of the pinhole (≈50 µm^[^ ^103^ ^]^), the size of the numerical aperture of the objective lens (typically 1.3–1.4^[^ ^105^, ^108^ ^]^), and the wavelength of the laser beam. Although, the image quality can be poor for quantitative measurement of cell dimensions at depths below 10–20 µm.^[^ ^103^, ^105^, ^107^ ^]^ A “*z*‐stack” of the images can be compiled to form a composite 3D image of a thick sample. While the theoretical axial *z* resolution is high (≈50 nm^[^ ^98^ ^]^), practical *z* resolution is >0.7 µm, two to four times poorer than the lateral *x–y* resolution.^[^ ^103^, ^105^ ^]^

The high resolution reconstructed 3D images achievable through CLSM, which include sub‐micrometer details such as torus‐margo bordered pit structures and oriented cellulose microfibrils, is substantially better than what can be obtained through micro‐CT scanning, though the latter can image larger volumes.^[^ ^105^, ^109^ ^]^ To reduce signal to noise ratio and achieve high‐quality images, using small *z*‐steps (e.g., 150 nm^[^ ^105^ ^]^), and averaging 8^[^ ^107^ ^]^ to 50^[^ ^105^ ^]^ scans at each *z*‐step optical section is recommended. Jang et al.^[^ ^105^ ^]^ report that scanning 128 optical sections 50 times to generate a 2 × 10^−5^ mm^3^ 3D image of a 20.5 µm wood fiber took under 30 s. As imaging speeds have improved, researchers have imaged larger field of views; for instance, 1024 × 1024 pixels square, corresponding to a 625 × 625 µm square, can be imaged at speeds of around 25 Hz,^[^ ^103^, ^110^ ^]^ with modern CLSM instruments approaching 200 frames s^−1^. Unsurprisingly, the principal limitation to the use of CLSM has been the large quantity of data generated, and the need for significant computing power for 3D reconstruction.^[^ ^93^, ^100^, ^101^ ^]^

**Figure 7:**
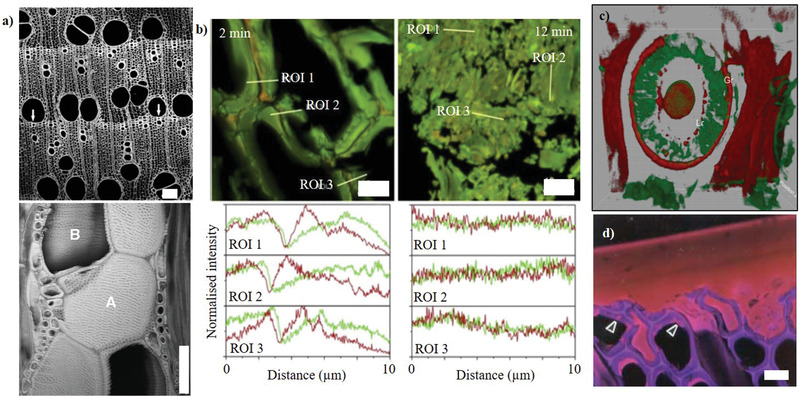
Confocal microscopy has found a variety of uses in wood research. a) 2D optical section of ring‐porous hardwood tissue (top), enabling 3D rendering of 61 tangential optical sections at 1 µm intervals. Vessels are labelled as A and B. 100 µm scale bars. b) Intensity of cell wall polymer distribution (lignin in red, and polysaccharides in green) measured across regions of interest (ROI) following short periods (2 or 12 min) of mechanical milling. Scale bar: 10 µm. c) 3D reconstruction of a bordered pit, with the greater and lesser rings (Gr, Lr) visible and pectin (red) and crystalline cellulose (green) are labeled. 36 µm × 36 µm square. d) Adhesive penetration. Scale bar: 25 µm. a) Adaptated with permission.^[^ ^106^ ^]^ Copyright 2004, Botanical Society of America, published by John Wiley and Sons. b) Reproduced with permission.^[^ ^122^ ^]^ Copyright 2017, Elsevier. c) Reproduced with permission.^[^ ^115^ ^]^ Copyright 2013, Botanical Society of America, published by Wiley. d) Adapted with permission.^[^ ^118^ ^]^ Copyright 2004, Springer Nature.

CLSM has become an incredibly versatile technique (**Figure** **7**). It has found a variety of uses in wood research,^[^ ^95^, ^96^, ^109^ ^]^ including to visualize 2D and 3D ultrastructure^[^ ^97^, ^98^, ^106^, ^107^, ^109^, ^110^, ^111^, ^112^ ^]^ (Figure 7a,c), measure cell dimensions,^[^ ^103^, ^105^, ^108^ ^]^ and determine the cellulose microfibril angle;^[^ ^113^, ^114^ ^]^ study cell wall polymer composition^[^ ^107^, ^109^, ^112^, ^115^ ^]^ (Figure 7a–c); inspect (the effects of) liquid penetration, including water^[^ ^116^, ^117^ ^]^ and polymers^[^ ^118^, ^119^ ^]^ (Figure 7d); and assess ageing and (bio)degradation (Figure 7b).^[^ ^120^, ^121^, ^122^ ^]^ Most of these ultrastructural observations can inform or aid discussion of structural properties of natural and/or modified wood. One notable example of this is the work of Bergander and Salmèn^[^ ^123^ ^]^ who explored the relationship between the microfibril angle and the transverse mechanical properties of wood tracheids (fibers). Extending the approach by Batchelor et al.,^[^ ^124^ ^]^ who developed an approach using CLSM and polarizing optics to obtain the microfibril angles of wood cell walls,^[^ ^124^ ^]^ they showed that while the microfibril angle in the S2 layer has a strong influence on the longitudinal stiffness of wood fibers, it has very little effect on the transverse properties.^[^ ^123^ ^]^ Greater influence was found to be exerted by microfibril angles in the S1 and S3 layers, something which would not have been understood without the use of CLSM.^[^ ^123^ ^]^ Another example is in helping us understand the fracture behavior of wood. Dill‐Langer et al.^[^ ^125^ ^]^ loaded wood specimens in different directions to the grain, and from earlywood and latewood sections, while following crack growth using CLSM.^[^ ^125^ ^]^ In this case, CLSM had advantages over other approaches, such as scanning electron microscopy, because it enabled samples to be tested without the need to evacuate a testing chamber, thereby drying out the specimen. Wood's mechanical properties are highly sensitive to moisture, so CLSM allows natural conditions of testing to be maintained.^[^ ^125^ ^]^ Two major fracture mechanisms were identified, which became dominant depending on the load direction. When load was applied parallel to the grain, wood cell to wood cell delamination fracture occurred in an almost brittle manner.^[^ ^125^ ^]^ However, perpendicular to the grain, cell wall fracture occurred and a stepwise crack emerged; the cell wall fracture was found to progress through areas of narrow cell wall thickness, i.e., earlywood sections.^[^ ^125^ ^]^ Specimens were also loaded at 45° to the grain direction, wherein fracture occurred according to a mixed mode between these two mechanisms.^[^ ^93^, ^100^, ^101^, ^103^, ^105^, ^107^, ^110^ ^]^ The flow of liquids into woods, and its effects on wood microstructure and properties has been studied using CLSM (Figure 7d). Conducting CLSM experiments in an environmental humidity chamber, with humidity changing between <30% and >80%^[^ ^116^, ^117^ ^]^ the anisotropic swelling and shrinkage behavior of tracheid cells during water adsorption and desorption has been visualized. The progressive imbibition and uptake of water through tracheid cells and how this is different in deformed tracheids was also studied, revealing that flow mainly occurred through the tracheids at the interface of deformed and undeformed regions.^[^ ^117^ ^]^ In contrast, CLSM confirmed the absence of tracer dyes in softwood ray cells suggesting that they do not have a role to play in radial water transport.^[^ ^127^ ^]^ Indeed, interactions of wood (and its constituent polymers) with water is critical in its natural processing (namely, self‐assembling of the cell wall polysaccharides), and water is a governing parameter influencing wood's mechanical properties. CLSM can be used as a tool to further inform us of the role of water. For example, CLSM can be used to examine microporosity distribution in wood cell walls, and to study bound water within the cell walls.^[^ ^128^ ^]^

To form structures and components with wood materials, apart from mechanical connections, adhesive bonding is popular. The mechanical performance of the adhesive bond is strongly related to wood‐polymer chemical affinity, and the wetting and impregnation of the polymer adhesive/resin into the wood microstructure.^[^ ^129^ ^]^ CLSM is well poised to study both the aspects and has been frequently used for this purpose, for a wide range of polymer adhesives, coatings, treatments and grafting.^[^ ^118^, ^119^, ^130^ ^]^ CLSM is often used in a complementary way to other microscopy techniques in this area of research. CLSM enables measurement of penetration depth, the identification of fluid transport pathways, the localization of polymer or cell wall modification (e.g., delignification), and even the quantitative visualization of which polymers can penetrate into the cell wall microporosity (Figure 7d). The latter can be complemented with micromechanical studies, such as nanoindentation tests, in which hardness and elastic modulus of cell walls, as a distance from the bond line, are measured^[^ ^119^ ^]^ Nanomechanical measurements using AFM have also been used in combination with optical imaging to map the structure of wood (see Section 7).

Not only is the principle of CLSM enticing for wood research, the necessary sample preparation (or lack of) can also be attractive. Sensitive wood material samples, such as fragile cell walls of differentiating xylem and even lignified fiber cells, can deform at the region of interest from physical sectioning, sample drying, and embedding processes.^[^ ^103^, ^105^, ^107^ ^]^ Since one can observe relatively thick samples, and visualize deep into the surface to obtain 2D optical sections and 3D volumetric images, CLSM avoids influence of any distortion and cell damage resulting from physical sectioning of such sensitive samples. CLSM also does not require the drying of samples.^[^ ^107^, ^110^ ^]^ Studying the distribution of aspirated bordered pits in green and air‐dried softwood, Matsumara et al.^[^ ^110^ ^]^ demonstrated that optical sections using CLSM could be produced for a range of wood moisture conditions (up to 250% moisture content), without “physical” sectioning (i.e., microtoming) or embedding of the wood sample, and in autofluorescence mode (i.e., with no dye staining necessary).^[^ ^103^, ^110^ ^]^ However, extremely fragile samples, such as 2000 year old water‐logged and buried timber,^[^ ^120^ ^]^ may need fixation in LR White resin (or similar) prior to any physical sectioning for imaging.^[^ ^120^ ^]^ Similarly, dye staining may be useful in, for example, visualizing the location of specific targeted cell wall constituents (e.g., cellulose and pectin), which do not autofluoresce, by enhancing their fluorescence signal. The different forms of sample preparation required is discussed below.

CLSM has been used to study different wood species (ring‐porous and diffuse‐porous hardwoods^[^ ^106^, ^107^, ^131^ ^]^ and softwoods like pine^[^ ^111^, ^112^, ^131^ ^]^) and tissue types (e.g., xylem,^[^ ^107^ ^]^ vessel,^[^ ^106^ ^]^ fibers,^[^ ^108^ ^]^ and cambium,^[^ ^109^ ^]^ reaction wood such as compression wood^[^ ^112^, ^114^ ^]^). Due to inherent differences in (auto‐)fluorescence in the tissues and the constituent polymers, sample preparation and conditions may require optimization.^[^ ^109^, ^131^ ^]^ Nakaba et al.^[^ ^109^ ^]^ and Donaldson^[^ ^131^ ^]^ review and discuss different sample‐protecting mounting media and fluorescence signal enhancing staining dyes for wood tissue.

Mounting media, typically water, glycerol solution, or oil, are used to prevent the wood sample from drying and to retard photobleaching during imaging. These can also act as clearing agents and enable deeper imaging. Usually a medium with a high refractive index, matching or close to that of the objective, are preferred. Several studies report oil and glycerol solution as more effective mounting media in comparison to water^[^ ^98^, ^103^, ^105^, ^109^, ^131^ ^]^ though swelling‐induced delamination for glycerol mounted specimen and hindered infiltration and presence air bubbles in immersion oil mounted wood specimens have been raised as potential issues.^[^ ^131^ ^]^

Wood cell walls are naturally fluorescent due to the presence of lignin. Lignin fluoresces over a broad range of wavelengths.^[^ ^108^, ^110^, ^112^, ^114^, ^131^ ^]^ Indeed, CLSM in autofluorescence mode can not only be used for visualizing ultrastructure, but also to assess lignin content distribution in wood.^[^ ^112^, ^131^ ^]^ For example, lignin autofluorescence signals are strongest from the lignin‐rich middle lamellae, and much weaker from the S3 layer in normal wood and reaction wood of radiata pine.^[^ ^114^ ^]^ Secondary walls of fibers emit less autofluorescence than walls of vessel elements.^[^ ^106^ ^]^ Lignin distribution within a cell wall layer can also be observed, such as uniformity or gradients in lignin concentration in S2 and S1 cell wall layers of radiata pine's compression wood, respectively.^[^ ^112^ ^]^ Advantages of autofluorescence CLSM include the simplicity of the process (i.e., a toxic stain and staining procedure is not necessary), and the ability to localize lignin in the image.^[^ ^132^ ^]^ However, changes in lignin composition and interference with other aromatic components can influence fluorescence intensity, making quantification difficult.

The fluorescence signal can be enhanced by staining samples with fluorochromic dyes. Nakaba et al.^[^ ^109^ ^]^ present detailed protocols for staining wood materials for specific investigations. In general, similar dyes to those used in wide‐field fluorescence can be used for CLSM.^[^ ^95^, ^96^ ^]^ Some dyes lack specificity and will stain a range of materials in plant tissue, for example, safranin, a commonly used dye, cannot differentiate between lignified and unlignified material, and will stain cellulose and resin alongside lignin.^[^ ^131^ ^]^ Acriflavin and berberine sulphate, like safranin, are examples of general stains lacking specificity. Typically, calcofluor white, but also congo red and pontamine fast scarlet 4B, is used to stain cellulose. Notably, cell walls stained with any of these cellulose‐affinity dyes exhibit bifluorescence when rotated on the microscope stage; as their fluorescence depends on orientation, they are useful in measuring cellulose microfibril orientation in cell walls.^[^ ^96^, ^113^, ^114^, ^131^ ^]^ Specific labeling of pectins and crystalline cellulose is also possible, as was done to study polymer distribution in aspirated and unaspirated torus‐margo bordered pit structures (Figure 7c).^[^ ^115^ ^]^ Dyeing of wood samples is typically carried out at room temperature in dilute solutions (0.001–0.1% in water or ethanol), with dyeing times ranging from a few minutes to over a day long, if drying is involved. Different wood species and tissue types are better observed through specific dyes based on their lignin and cellulose content. In addition, these fluorescent dyes excite over a specific range of wavelengths and hence the laser beam needs to be appropriately selected.^[^ ^131^ ^]^

To complement observations in fluorescence mode, CLSM can also be run in reflected light mode.^[^ ^105^, ^111^ ^]^ Light is reflected from surfaces, boundaries and interfaces of two media with different refractive indices. Consequently, a reflection mode is ideal to study wood surface features, such as fiber surfaces and microfibril angle.^[^ ^95^, ^96^ ^]^

## 7 Atomic Force Microscopy Imaging and Nanomechanics of Wood

**Figure 8:**
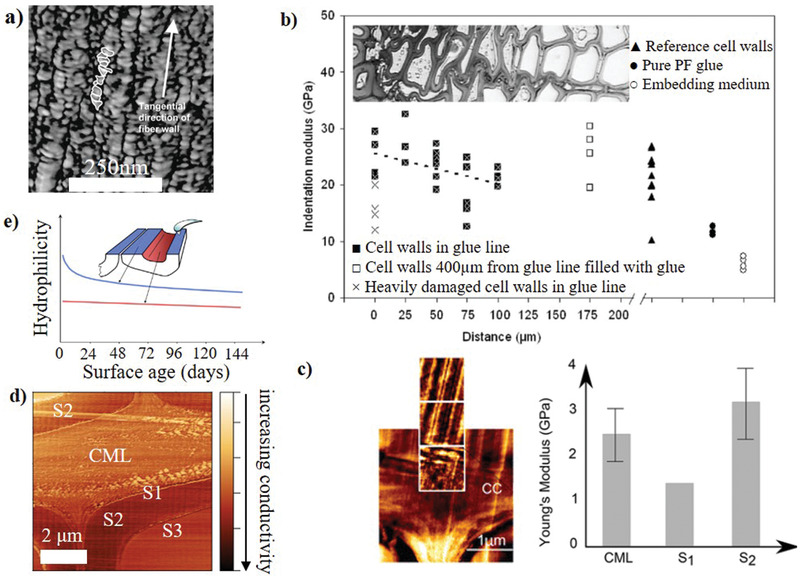
AFM has enabled imaging cell wall ultrastructure, as well as directly relating structure to bio‐chemical, mechanical and thermal properties. a) Imaging individual cellulose aggregates in the S2 layer. b) Spatial distribution of indentation modulus of cell walls at the adhesive bond line to study resin penetration into cell walls. c) Topological image of a cell wall junction which was scanned in AFM mode to measure differences in Young's modulus of cell wall layers. d) Imaging differences in thermal conductivity of cell wall layers of Norway spruce, relating to the different microfibril angle and cellulose to noncellulosic polymer ratio of each layer; CML is the central middle lamella. e) Imaging differences in polarity of the inner S3 layer and transverse sections S2 layer, as a function of time in days. a) Adapted with permission.^[^ ^135^ ^]^ Copyright 2003, Springer Nature. b) Adapted with permission.^[^ ^145^ ^]^ Copyright 2004, Elsevier. c) Adapted under the terms of the Creative Commons Attribution 4.0 International License (https://creativecommons.org/licenses/by/4.0/).^[^ ^140^ ^]^ Copyright 2017, The Authors, published by BioMed Central, part of Springer Nature. e) Adapted with permission.^[^ ^148^ ^]^ Copyright 2014, Elsevier.

Atomic force microscopy (AFM) is a scanning probe microscopy technique wherein a probe tip is rastered over the sample surface, and the vertical motion of the tip is measured by the reflection of a laser beam from the top surface of the probe into a photodetector (**Figure** **8**). Ever decreasing AFM probe tip sizes (<10 nm) produce topographical images with high *x*–*y* resolution (of tens of nanometers) and ultrahigh *z*‐resolution (<0.1 nm). The latter is several orders of magnitude better than resolutions achievable through diffraction‐limited optical microscopy. Consequently, AFM has been effectively used to visualize plant cell wall layers^[^ ^133^, ^134^ ^]^—particularly primary and S2 layers—as well as the architecture of these layers, such as the arrangement, orientation, and size (length and diameter) of cellulose microfibrils^[^ ^134^, ^135^ ^]^ (Figure 8a) and the distribution of pore sizes.^[^ ^136^ ^]^ Several reviews have discussed the progress in plant cell wall research that AFM has enabled.^[^ ^137^, ^138^ ^]^ AFM is inherently dependent on probe–surface interactions, and therefore probe geometry (size and shape) and sample surface roughness plays a significant role in establishing the image quality and resolution.^[^ ^138^, ^139^ ^]^ Preparation of smooth samples may require ultra‐microtoming with a diamond knife, although thicker samples can be used. To reduce damage to cell walls during sectioning, embedding wood samples in resins is common, though not necessary.^[^ ^140^ ^]^

The real beauty with AFM lies in the possibility to modify the probe tip to simultaneously image wood cell wall ultrastructure and measure cell wall properties (mechanical, chemical or thermal), enabling direct elucidation of underlying structure–property relations (Figure 8).

The use of a cantilevering sharp tip with a known spring constant enables nanoindentation studies at nN force resolution across the entire sample surface (Figure 8b,c). This maps the distribution in elastic modulus (stiffness and indentation hardness) and viscoelastic response of different cell wall regions (early wood, late wood, and reaction wood) and cell wall lamella, including in relation to cellulose microfibril orientation and arrangement^[^ ^138^, ^140^, ^141^, ^142^ ^]^ and lignin distribution.^[^ ^142^ ^]^ Reduction in indentation modulus with increasing MFA and Young's modulus of layers in the order of S2 > CML > S1 are typical observations (Figure 8c).

The indentation modulus obtained from such nanomechanical studies is not, however, a measure of (or directly comparable to) the longitudinal elastic modulus of a wood cell wall (layer), as the transverse modulus of the cell wall (layer) also plays a role on the indentation response.^[^ ^143^ ^]^ In addition, it should be noted that while nanomechanical AFM measurements are useful for relative comparison of cell wall properties within a study, cross‐study comparisons are not easily possible as the measured values depend on calibration parameters (e.g., properties of the embedded resin), assumed mechanical models, presence of artefacts, and quality of sample preparation.^[^ ^140^, ^144^ ^]^ Nonetheless, more AFM studies are needed to examine the influence of cell types, wood species, and drying and moisture content on the mechanics of indentation.^[^ ^140^ ^]^

AFM has been used to examine how cell wall molecular architecture may change through processing; for example, broadening of pore sizes and enlargement of microfibrils was observed during the chemical pulping of wood.^[^ ^135^, ^136^ ^]^ Nanomechanical AFM experiments have also been used to examine the penetration of polymer adhesives into wood cell walls and assess consequent changes in cell wall and interphase mechanical properties.^[^ ^145^ ^]^ Indeed, the preferential adhesion of an impregnated polymer to a specific cell wall layer can also be measured; for example urea formaldehyde adhesives show better adhesion to the more hydrophilic S2 layer than to the inner cell wall surface of the S3‐layer (Figure 8e), while polyurethane adhesives show the opposite trend.^[^ ^146^ ^]^

Aside from unraveling relations between cell wall structure and mechanical properties, AFM may also be used to relate cell wall structure to thermal properties and chemical properties, through scanning thermal microscopy (SThM) and chemical force microscopy (CFM), respectively. A SThM probe, which acts as a resistive heater, when attached to the AFM enables mapping thermal conductivity variations across the wood ultrastructure (Figure 8d) and can relate this to its anatomical organization. SThM studies on wood have been fruitful in revealing ultrastructural information (e.g., orientation of cellulose microfibrils in different cell wall layers owing to anisotropic conductivity of cellulose fibrils),^[^ ^149^, ^150^ ^]^ monitoring adhesive penetration at a bond line,^[^ ^151^ ^]^ and assessing the effects of carbonization between 200 and 600 °C on wood microstructure (e.g., wall thickness) and composition.^[^ ^152^ ^]^

As AFM relies on probe–surface interaction, adhesion forces between the probe and the surface can be mapped by either chemically functionalizing the AFM tip and/or functionalizing the wood sample (i.e., chemical force microscopy (CFM)).^[^ ^148^, ^153^ ^]^ These adhesion force maps can then be used to infer surface polarity and chemical properties of the wood cell walls.^[^ ^148^, ^153^ ^]^ Indeed, impregnated or chemically modified wood samples can be probed with polar and nonpolar functionalized tips to reveal the precise location and polarity/hydrophilicity of the introduced modification/functional groups (Figure 8e). As with nanomechanical AFM studies, more scientific studies probing wood with SThM and CFM and other such techniques are necessary to appreciate and realize the full potential of these scanning probe microscopy techniques.

## 8 Discussion and Future Perspectives

**Figure 9:**
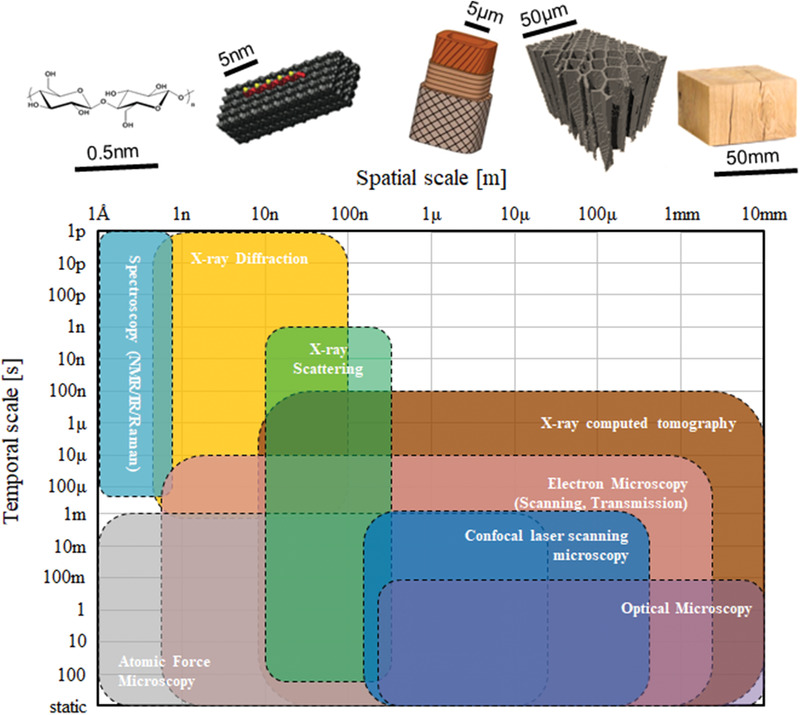
The range of spatial and temporal scales enveloped by the various spectro‐microscopy techniques in imaging woods (and similar plant‐based materials). The relevant length scales of wood's hierarchical structure are presented for reference. Figure drawn using data from refs. ^[^ ^158^, ^159^ ^]^.

This review has shown that wood, because of its hierarchical structure and complexity, both lends itself to techniques that span both length and temporal scales, but also that it necessitates such analyses to understand structure–property relationships. In this sense, wood could be regarded as both an ideal and challenging material for imaging characterization techniques. The range of techniques covered in this review are summarized with regards to limits of their spatial and temporal scales in **Figure** **9**, which reflects these ranges seen in wood. The spatial scale reflects the length scale(s) reliably observable through the technique, and is limited by the (practical) resolution, and size of scanned region or specimen size limit. For example, X‐ray tomography can be used to examine timber at a very fine resolution (≈10 nm), and can be programmed to scan fairly large samples (exceeding 10 mm). The temporal scale reflects the time scale(s) reliably observable by the technique, and is limited by the temporal resolution or speed/rate of imaging or data acquisition, and the time period over which images or data can be collected. The reviewed techniques can probe length scales from ≈1 Å (e.g., spectroscopy techniques such as NMR, FTIR and Raman, and AFM) to tens of millimeters (optical microscopy and X‐ray CT), and over time scales of less than 1 ps to (quasi‐)static. These techniques enable visualization or measurements ranging from very fast molecular‐scale interactions between cell wall polymers to very slow macroscale evolutions in the tissue structure. Combining complementary techniques, such as confocal Raman, can help cover a wider range of spatial and temporal scales. Nonetheless, a notable “gap” in imaging techniques is at the top‐right quadrant, with large spatial scale but small temporal scale.

A thorough understanding of wood structure from the nano‐ to the macroscale can be achieved through an experimental program that combines different microscopic and spectroscopic techniques. Such an experimental program will not only cross‐validate experimental findings but also complement results where the limitations of one method can be overcome by the advances of the other. The effect of variations in sample preparations among methods can be better understood when various techniques are compared. Yet, the required time investment and the level of expertise needed in such studies can be a deterrent to use despite the richness of information achieved. The need for interdisciplinary research for an in depth understanding of wood's response to mechanical and environmental conditions is reflected in recent studies on the lateral deformation of wood under compressive loading^[^ ^154^ ^]^ and on the chemical degradation of waterlogged archaeological wood (WAW).^[^ ^155^ ^]^ In the former study a cell wall polymer redistribution was reported in densified softwood at ambient temperature. Higher lignin and cellulose concentration was observed in the compressed areas of the cell walls using CRM and TEM and confirmed based on the Young's modulus values from correlative Raman/AFM. A thorough study on waterlogged archaeological wood^[^ ^155^ ^]^ employed the use of SEM, ATR‐FTIR and Raman imaging, NMR, WAXS, and nitrogen adsorption studies. Although SEM images showed relatively intact cell walls in samples from a 170 year old shipwreck, a decrease in both hardness and stiffness was reported with AFM. Wet chemical analysis and FTIR and NMR studies suggested the degradation of hemicellulose and cellulose. A decreased cellulose crystallinity was confirmed in WAW compared with raw samples using WAXS and lignin alteration combined with a decrease in unconjugated LCC ester linkages was observed through Raman imaging as also inferred via the NMR and FTIR studies.

Optical sectioning is the main advantage of confocal microscopy over other optical, scanning electron and transmission electron microscopy techniques, though confocal microscopy should be thought of and used as a complementary technique, not as a substitutive technique.^[^ ^95^, ^98^, ^103^, ^105^, ^109^ ^]^ The quality of confocal images from wood samples, ranging from translucent 20–100 µm microtomed sections to 1–2.5 cm thick hand‐sectioned pieces, is comparable to images of Raman spectroscopy,^[^ ^156^ ^]^ atomic force microscopy,^[^ ^157^ ^]^ confocal fluorescence resonance energy transfer (confocal FRET) microscopy,^[^ ^128^ ^]^ and 4Pi microscopy.^[^ ^115^ ^]^ Of these, confocal Raman spectroscopy is the most popular, as it is very compatible with CLSM and enables detailed evaluation of chemical composition and molecular cell wall organization with high spatial resolution. CLSM can be further used to observe ultrastructural changes and alterations in lignin and polysaccharide distribution in cell walls when wood is disintegrated in mechanical processes, such as milling.^[^ ^122^ ^]^ Complementary TEM and XRD can reveal further information regarding the deconstruction of wood cell walls. Solid‐state ^13^C NMR combined with WAXS^[^ ^50^ ^]^ and FTIR spectroscopy^[^ ^160^ ^]^ have been adopted to study cellulose structure and crystallinity though WAXS has been proven to better detect cellulose crystallinity.^[^ ^161^ ^]^ Cellulose conformations and cellulose orientation and chain arrangement within the microfibril are accurately identified with ^13^C‐NMR and WAXS, respectively.^[^ ^50^, ^160^ ^]^ FTIR is more suitable for the hydrogen bonding interactions between cellulose chains within the microfibril.^[^ ^50^ ^]^

There are several other issues with, and properties of wood, that necessitate the various techniques used, but also require more research to better understand properties at the various length scales. It is well known that the properties of wood are highly variable, depending on species, location of the samples within the structure, and also the environmental conditions under which the wood is grown and/or tested. Little standardization is applied to the preconditioning of wood, both in the growth stages, nor in the testing protocols employed across the various techniques used. It would be useful to carry out a “Round‐Robin” style testing of wood, with standard samples preconditioned and grown from the same initial species, using the various techniques that we have reviewed. This type of exercise, primarily using different mechanical testing and not imaging methods, has been employed in the past for more conventional composite materials.^[^ ^162^ ^]^ Given the range of mechanical properties exhibited by wood, and that these properties are often a result of the hierarchical structural differences, such a testing exercise could be very instructive to understanding not just the material variability, but variations between laboratories. Limited microscopic and spectroscopic studies can accommodate a mechanical testing device (e.g., SEM and X‐ray diffraction) and data acquisition time, resolution and sample size can often be a challenge. Data acquisition times are critical for the proper interpretation of the experimental results given that wood is a viscoelastic material and its mechanical properties are highly dependent on strain‐rate and creep effects but also moisture content. Studies that relate mechanical properties to the MFA and wood polymer components (e.g., through stretching of molecular bonds upon external strain^[^ ^72^ ^]^) are well established. Yet, there is limited understanding on how the spatial interaction and linking of the wood polymer components within each cell wall and between cell wall layers and their different structural forms (e.g., crystalline and noncrystalline cellulose) affect their mechanical and physical properties and if a single cell wall model can be adopted for both primary and secondary walls in all wood species.^[^ ^3^ ^]^ A “stick‐slip” mechanism between cellulose microfibrils (hydrogen bonding between hemicellulose and cellulose) has been suggested to elucidate the stiffness properties of wood^[^ ^40^, ^163^ ^]^ and fracture failure modes have been reported in the middle lamella and S1/S2 layer due to abrupt changes in lignin concentrations.^[^ ^164^ ^]^ Several models have proposed that the viscous properties of wood are related to the lignin‐hemicellulose matrix component in the secondary walls^[^ ^165^ ^]^ and that the activation energy of failure slip planes in wood is related to breakage of hydrogen bonds within cellulose^[^ ^166^ ^]^ but also possibly between hemicellulose and cellulose. Yet, how the degree of crosslinking between the wood polymer components affects mechanical properties is still not clear. It has been suggested that the decrease in the number of crosslinks in xyloglucan/cellulose composites results in a stiffer and stronger mechanical performance and higher creep resistance closer to cellulose composites.^[^ ^167^ ^]^ The effect of fiber entanglement at higher strains should also be considered. Another challenge is to understand the hierarchical and percentage contribution of each wood structural component to the physical and mechanical properties. Does the cellular structure and MFA play a greater role in the wood performance than the spatial and chemical interactions between wood polymer components? Given the anisotropic performance of wood it is expected that different components contribute to different mechanical properties. Integrated imaging and mechanical screening test methods combined with molecular dynamics simulations in both native and modified wood could potentially improve our knowledge on these areas.

New advances in imaging are needed to perhaps overcome some of the issues mentioned above, to simultaneously record the chemical and spatial make‐up of the cell walls of wood, with advanced optics to record signals much more rapidly. More rapid acquisition times have been achieved in recent times with Raman and infrared spectroscopies, and the use of microfocus and sub‐micrometer focus X‐ray beamlines (e.g., at the European Synchrotron Radiation Facility) have enabled much greater detail to obtained at smaller length scales of the wood cell wall. Recent advances in microscopy with the use of an helium ion source have revealed the ultrastructure of *Arabidopsis* at higher resolution and greater depth of field compared with FE‐SEM.^[^ ^168^ ^]^ Some of the advantages of Helium Ion microscopy are the lack of heavy metal coatings for sample preparation and the minimization of sample damage from beam irradiation. In conjunction with these techniques, mechanical testing can be incorporated, or combinations of techniques (Raman/X‐ray) to enable greater depth and breadth of measurement (molecular/crystalline deformation) of wood specimens.

Tomographical imaging now allows larger components to be fully imaged, something which has been developed primarily for conventional industrial components, but could easily be used to image in‐service wood used in civil engineering construction projects. Reynolds et al.,^[^ ^16^ ^]^ in an effort to study fluid flow in softwood timber, successfully drew correlations between micro‐computed X‐ray tomography measurements at two different resolutions. Low‐resolution tomography on structural‐size pieces of wood revealed variations in porosity across the wood's structure, while detailed though time‐consuming high‐resolution tomography on matchstick size specimens informed precise cell morphometry (cell length, wall thickness, and lumen area). This study demonstrated that data from the low‐resolution scans could estimate the variation in (small‐scale) cell geometry throughout a structural‐size piece of wood. Burridge et al.,^[^ ^169^ ^]^ in their follow‐on study, utilized these parametrized data on timber microstructure (particularly pore space) to accurately predict time‐dependent fluid ingress and flow in timber. The use of such low‐resolution X‐ray tomography on structural pieces of timber (or even standing trees) may help further the digitization of the timber industry in grading wood (in terms of its quality and mechanical properties), as well as efficiently modifying its properties (through optimized impregnation with chemicals and polymers). Such tomographic data may also help visualize and better model the fracture behavior and mechanisms of wood (e.g., earlywood and latewood failures), when subjected to specific loading conditions.

Another major issue concerning wood is the effect of sample size on mechanical properties. As has been demonstrated, each of the imaging techniques requires a certain sample size, be it a thin section for Raman and IR imaging, to cubes and blocks of material required for X‐ray tomography. The question naturally arises as to whether such sample sizes are representative of the bulk properties of wood, i.e., do they incorporate enough material to reflect inherent variability? Given these limitations it is also questionable that there is a limit to how well these model specimens can truly inform molecular models of the structure of the cell wall. Moreover, further understanding on how to extrapolate research on plant dicot models, such as *Arabidopsis*, to woody dicots is needed. Variations between the structural arrangement of xylem and the degree of lignification lie between herbaceous and woody dicots. However, plant models can be easily genetically modified and provide useful information on the physical and mechanical properties of wood polymer components and their backbone decorations (e.g., acetyl groups in xylan). Yet, there is still ongoing research in genomic methods for plants (including *Arabidopsis*) to characterize all enzymes related to genetic and enzymatic processes.^[^ ^170^ ^]^ In general, the reviewed spectro‐microscopic techniques will continue to be useful tools in plant biomechanics and phenotyping studies. Genetically modified plants have exhibited increased amounts of the remaining wood polymer components to compensate for the deficiency of a particular polymer. Mutations that affect cellulose synthesis can lead to increased amounts of pectin in the primary cell walls^[^ ^170^ ^]^ and an increase in the microfibril diameter has been reported in xyloglycan deficient *Arabidopsis* primary cell walls.^[^ ^171^ ^]^ *Arabidopsis* plants are preferred for genetic modifications due to their small genome and short lifespan.^[^ ^172^ ^]^ However, transgenic aspen trees with suppressed Pt4CL1 expression that is related to lignin biosynthesis have exhibited similar cell wall structure with wild type and unaltered lignin structure^[^ ^173^ ^]^ in contradiction with other negative effects observed in herbaceous plants.^[^ ^173^, ^174^ ^]^ In^[^ ^173^ ^]^ a pronounced reduction in lignin content was compensated with an increase in cellulose, as also observed in tension wood, and the enhanced growth was attributed to modifications in the complex signaling during growth and increased carbon supply as a result of lignin reduction. Genetic modification studies that relate to lignin alteration and reduced lignin content are of interest to reduce lignocellulose recalcitrance and increase biomass.^[^ ^175^, ^176^ ^]^ CAD‐downregulated poplar trees with reduced lignin content and increased hydroxycinnamaldehyde incorporation have shown decreased tensile Young's modulus and tensile strength.^[^ ^176^ ^]^ Minor variations in the wood density and MFA were detected between the transgenic and wild type samples and different lignin deposition in the middle lamella and secondary wall with intact cellulose and hemicellulose structure were reported in the mutants, as observed via a complementary study with WAXS, FTIR and Raman spectroscopy.^[^ ^176^ ^]^ Knowledge of the molecular architecture of the cell wall will inform models and enable us to breed wood species tailored to specific needs such as better mechanical resistance and durability and biomass with reduced recalcitrance. Molecular cell wall studies in both native and modified wood can improve current modification processes and extend them to industrial scales. Moreover, cell wall molecular dynamic simulations based on well informed input data can be a powerful tool to optimize wood modification treatments tailored to specific requirements avoiding tedious and time‐consuming experiments. This is critical before commercialization and timber modification at greater industrial scale takes place.^[^ ^177^ ^]^

Most current treatments to modify wood properties are derived from scientific experiments or historical observations of old manufacturing methods. Current studies on the cell wall molecular architecture of modified wood aim to understand wood chemistry related to the experimentally observed physical, mechanical and biological properties. In 1920, Tiemann observed that drying of wood at high temperature results in decreased hygroscopicity and greater dimensional stability.^[^ ^178^ ^]^ Heat treated wood also exhibits improved resistance to biological degradation and a rise in stiffness and strength can be observed when the process is carried out in a vacuum or in air.^[^ ^179^ ^]^TH treatments combined with compression of wood in the transverse direction causes densification by reducing the void volume of the cell walls. An increase up to 41% and 45% has been reported in the modulus of rupture and modulus of elasticity, respectively, for poplar densified in this way.^[^ ^180^ ^]^ However, hot pressing after chemical treatment to partially remove lignin/hemicellulose can lead up to 11.5 times rise in the tensile strength of basswood.^[^ ^20^ ^]^ Chemical modification of wood either with the acetylation or a furfurylation procedure leads to improved biological durability, greater hydrophobicity and hardness.^[^ ^181^ ^]^ Wood welding through mechanical friction leads to chemical and physical modification of the wooden surfaces creating bond lines of equivalent strength to those derived from conventional commercial wood adhesives.^[^ ^182^ ^]^ Complementary imaging techniques have enabled a better understanding of these processes taking place but yet combined mechanical and imaging experimental protocols are necessary to optimize their effectiveness, and ensure safety when used in construction. Recent studies on wood composites with Metal Organic Frameworks (e.g., zeolitic imidazolate framework‐8 (ZIF‐8)^[^ ^183^ ^]^) and CaCO_3_^[^ ^184^ ^]^ have demonstrated the importance of adopting complementary imaging techniques. These assist an understanding of the effectiveness of pretreatment methods, chemical deposition, and distribution of additives and how this is reflected in the mechanical properties. In previously published work^[^ ^183^ ^]^ beech composite materials with zeolitic imidazolate framework‐8 have exhibited 47% higher compressive strength than native samples but yielded lower compressive modulus due to the partial degradation of lignin and hemicellulose in the pretreatment stage. The differences in the tensile modulus were less pronounced. The higher surface area of the ZIF‐8 resulted in a composite with increased CO_2_ adsorption capacity. Composites with CaCO_3_ have proven promising for potential applications as fire resistant engineered wood products.^[^ ^184^ ^]^

One major area of development for timber products in construction is its modification and the use of glue lines and welding/melding processes to generate greater spans and enhance mechanical function. Spectroscopic and microscopic studies have been very informative regarding the cell wall structure of current wood modification methods. Stamm et al.^[^ ^185^ ^]^ distinguished the bond line of welded wood specimens into an amorphous and a densified zone using CLSM. SEM microscopy on the welded surface of beech specimens in^[^ ^186^ ^]^ revealed entanglement of long wood fibrillar structures into a mass of molten polymer. Serious degradation of wood cells was not observed, and melting was postulated to be concentrated in the lignin rich middle lamella area. The matrix component of the bond line was confirmed to be lignin‐based with small amounts of hemicellulose as derived from CP MAS ^13^C‐NMR spectroscopy studies. Pizzi et al.^[^ ^187^ ^]^ identified an additional crystalline structure of the carbohydrates, observed in the matrix component of the bond line. This structure was attributed to either the presence of short cellulose fibrils from the degradation of the crystalline cellulose at the developed high temperature of friction welding or to recrystallization of hardwood xylan, as confirmed by SEM. A high degree of lignin demethoxylation and deacetylation of hemicellulose leading to the formation of furanic compounds have also been observed^[^ ^186^ ^]^ during wood welding. The latter led to furfural self‐polymerization and their possible linking with lignin aromatic rings. Lignin autocondensation was also dominant. The observed modifications in the wood chemical components during friction welding are related to the wood transformation at high temperature. Solid‐state ^13^C‐NMR studies have shown that TH treatments result in formation of acetic acid from hemicellulose leading to carbohydrate and lignin cleavage, lignin demethoxylation and formaldehyde, furfural and other aldehyde production.^[^ ^188^ ^]^ The availability of reactive sites in lignin leads to self‐crosslinking and additional lignin‐cellulose linking that is enhanced with a secondary curing treatment. The resulting “sheathing” of cellulose results in reduced hygroscopicity and dimensional stability.^[^ ^188^ ^]^ On the other hand, acetylation of wood through substitution of the hydroxyl groups with acetyl groups can also be an effective way of introducing hydrophobicity in wood. It has been demonstrated with both NMR and IR studies that lignin hydroxyl groups are acetylated first, with phenolic groups being the most reactive, followed by the free hydroxyl groups in noncrystalline regions of cellulose and xylan.^[^ ^189^ ^]^ Popescu et al.^[^ ^177^ ^]^ identified with IR spectroscopy three types of hydrogen bonded water in birch wood. In acetylated birch wood an increasing moisture content resulted in a decrease in the bound water with one engaged hydroxyl group. Regarding resin impregnated wood Nishida et al.^[^ ^190^ ^]^ could detect the reactivity and penetration of melamine formaldehyde (MF) and phenol formaldehyde (PF) resin in modified Japanese cypress based on NMR spectra and SEM images. PF resin reacted preferentially with amorphous cellulose at both the surface and intercellular regions of the cells. SEM, FTIR and Raman spectroscopy studies were employed^[^ ^191^ ^]^ combined with water uptake studies to investigate the efficiency of specific functionalization of Norway spruce by grafting polystyrene with the use of two methacryl precursors. Samples with a methacryloyl chloride precursor exhibited a thin layer of polystyrene (up to 3 µm) and higher water uptake compared with methacrylic anhydride samples that had a more uniform polystyrene distribution in the wood cell walls. All these physical and chemical modifications of wood require high temperature and are highly energy demanding, this detrimentally affecting the embodied carbon of engineered wood products. A thorough understanding of their mechanisms in the cell wall molecular architecture and subsequent genetic wood modification can be a more environmentally friendly way of producing wood with increased hydrophobicity, dimensional stability and biological durability. We expect that the use of such spectroscopic and microscopic techniques in the development of modified timbers will grow with the increased use of timber as a sustainable construction material.^[^ ^192^ ^]^ Some interesting examples may include functionalizing wood cell wall polymers with mechanophores and hydrophores to use as in situ strain or moisture sensors, and using imaging techniques (such as fluorescence resonance energy transfer microscopy) for nondestructive testing and maintenance of timber structures.

Imaging wood will help imagine new functionalities and properties. To realize this, collaboration between different disciplines (e.g., biochemists, chemists, material scientists, and engineers) will be necessary to bridge knowledge gaps from nano‐ to building scale. While we have used wood for millennia, we still do not fully understand how wood forms or is structurally arranged, and this knowledge, vital to realizing the full potential of timber, will only unravel by looking beyond what meets the eye in wood.

## Conflict of Interest

The authors declare no conflict of interest.
